# Biomarkers to Monitor Gluten-Free Diet Compliance in Celiac Patients

**DOI:** 10.3390/nu9010046

**Published:** 2017-01-06

**Authors:** María de Lourdes Moreno, Alfonso Rodríguez-Herrera, Carolina Sousa, Isabel Comino

**Affiliations:** 1 Departamento de Microbiología y Parasitología, Facultad de Farmacia, Universidad de Sevilla, c/Profesor García González 2, 41012 Sevilla, Spain; lmoreno@us.es (M.d.L.M); csoumar@us.es (C.S.); 2 Unidad de Gastroenterología y Nutrición, Instituto Hispalense de Pediatría, 41013 Sevilla, Spain; alfonsorodriguez@ihppediatria.com

**Keywords:** celiac disease, gluten-free diet, gluten immunogenic peptides, feces, urine

## Abstract

Gluten-free diet (GFD) is the only treatment for celiac disease (CD). There is a general consensus that strict GFD adherence in CD patients leads to full clinical and histological remission accompanied by improvement in quality of life and reduced long-term complications. Despite the importance of monitoring the GFD, there are no clear guidelines for assessing the outcome or for exploring its adherence. Available methods are insufficiently accurate to identify occasional gluten exposure that may cause intestinal mucosal damage. Serological tests are highly sensitive and specific for diagnosis, but do not predict recovery and are not useful for follow-up. The use of serial endoscopies, it is invasive and impractical for frequent monitoring, and dietary interview can be subjective. Therefore, the detection of gluten immunogenic peptides (GIP) in feces and urine have been proposed as new non-invasive biomarkers to detect gluten intake and verify GFD compliance in CD patients. These simple immunoassays in human samples could overcome some key unresolved scientific and clinical problems in CD management. It is a significant advance that opens up new possibilities for the clinicians to evaluate the CD treatment, GFD compliance, and improvement in the quality of life of CD patients.

## 1. Introduction

Gluten is a complex mixture of water insoluble proteins from wheat, barley, rye, and oats that are damaging to celiac patients. The term gluten includes prolamins (gliadins in wheat) and glutelins (glutenins in wheat). The prolamins, a complex group of alcohol-soluble proteins, constitute the major seed proteins in cereals and comprise about 50% of the proteins in mature cereal grain. Other gluten proteins showing that analogous immunogenic properties are present also in barley (hordeins), rye (secalins), oats (avenins), and other-closely related grains [1,2,3]. These proteins are rich in proline and glutamine residues, making them resistant to gastrointestinal digestion and encouraging the deamination by tissue transglutaminase (tTG).

Celiac disease (CD) is an immune-mediated systemic disorder elicited by ingestion of gluten in genetically-susceptible individuals. It affects around 1% of the population and it is based on a variable combination of intestinal and extraintestinal signs and symptoms, celiac specific antibodies, HLA-DQ2/DQ8 haplotypes, and enteropathy.

To date, the mainstay of CD is a lifelong strict gluten-free diet (GFD). There is a general consensus that strict GFD adherence in CD patient results in complete histological and clinical remission and an improvement in the quality of life and reduced long-term complications [4,5,6]. Thereby, the strict adherence to GFD leads to significant improvement in bone density [7,8,9,10] and the normalization of vitamins (e.g., vitamin B12 among others) and minerals, although sometimes supplements may be necessary to achieve optimum levels [11]. 

The gluten content in food is regulated by the Codex Alimentarius [12]. This regulation (CODEX STAN 118—1979, revised in 2008) states that gluten-free foods are those in which the total levels of gluten are ≤20 ppm [3]. Gluten-free cereals, such as rice, buckwheat, corn, and millet, can replace gluten-containing cereals. Some legumes, such as amaranth, quinoa, and soybean, are especially convenient due to their high protein content and quality. Moreover, non-processed food as fish, meat, poultry, egg, vegetables, and fruit are recommended to promote GFD adherence and secure the nutritional value of the diet [13].

Although conceptually simple, dietary changes are substantial and have a profound effect on a patient's life. Indeed, there are barriers related with GFD, such as availability, cost, and safety of gluten-free foods, or gluten cross-contamination [14,15]. Estimated compliance rates vary considerably (17%–80%), depending on factors such as the patient’s age or the age at diagnosis of CD, among others [16,17,18,19]. The poor dietary adherence has shown to be negative to promote other autoimmune disease [20,21], fertility problems [22,23,24], and increased risk of bone fracture [25] or lymphoma [26,27]. Furthermore, after adoption of the GFD, 4%–30% of CD patients reported persisting symptoms and are considered to be affected by nonresponsive CD (NRCD) [6]. However, only 10% of these NRCD patients have refractory CD (RCD), being inadvertent or deliberate gluten exposure the most frequent cause of NRCD [28].

Additionally, in the last decade CD research is changing rapidly as gluten-related disorders have gradually emerged as an epidemiologically-relevant phenomenon with a global prevalence. Such disorders include, besides CD, wheat allergy, which affects 0.2%–0.5% of the population [29], and non-celiac gluten sensitivity (NCGS), a pathology in which gluten ingestion results in symptomatic and morphological manifestations in the absence of CD and wheat allergy [30], with highly variable incidence from 0.6% to 6% [31]. It has become more complex both the differential diagnosis and monitoring of patients since the requirements of adherence to GFD vary in each of the disorders. Moreover, this also makes more noticeable the dilemma of how to measure dietary transgressions. Although the importance of monitoring the GFD, there are no clear guidelines for assessing the outcome or for exploring its adherence. In addition, there is no consensus on the frequency of monitoring or the suitable measurements for evaluating compliance and outcome [32]. A variety of surrogate markers are available to assess the GFD compliance including clinical assessment of symptoms, patient self-report about the level of adherence, dietary history, evaluation carried out by a professional nutritionist, small-bowel biopsy, or serologic screening tests. Nevertheless, the lack of standardized and accurate indicators of GFD adherence is a significant problem both in the clinic and in research.

In order to evaluate the recent literature relating to CD and the monitoring of GFD, a search of scientific literature was conducted for recent publications on GFD compliance and CD. Based on these updates, the aim of this paper is to show and discuss the current concepts on the available tools to follow-up patients on GFD.

The search was conducted in PubMed MEDLINE and SCOPUS databases. The following search terms were used: “celiac disease and gluten-free diet”, “follow-up celiac disease”, “monitoring gluten-free diet”, and “management celiac disease”. The keywords “symptoms and celiac disease”, “biopsy and celiac disease”, “serological test and celiac disease”, “questionnaire and celiac disease”, “dietary interview and celiac disease”, “feces and celiac disease”, and “urine and celiac disease” were also used.

## 2. Monitoring of Gluten-Free Diet Compliance

### 2.1. Symptom Assessment

Follow-up of initial symptoms or the manifestations of newly-developed ones serve to check the improvement and evolution of CD. Intestinal bowel symptoms have been reported as common in CD patients not adherent to GFD (odds ratios 2.69; 95% confidence intervals 0.75–9.56) according to a meta-analysis of seven studies, including more than 3000 subjects [33]. Although seemingly intuitive, clinical response could not be a single method for monitoring adherence to the GFD as a large number of celiac patients are asymptomatic or minimally symptomatic at presentation and in these cases it would not be feasible to use clinical response as an indicator of mucosal healing and GFD compliance [34]. A controlled study examining the effects of gluten challenge found that symptoms were absent in 22% of celiac patients, despite the presence of significant villous atrophy in the small bowel biopsy [35].

### 2.2. Validated Surveys and Dietary Interviews

The dietitian or dedicated physician is responsible for dietetic review. In addition to a number of questionnaires evaluating food frequency and self-reported GFD adherence, there is a visual analogue score scale which consists of an unmarked line with the anchor sentences ‘I never adhere to my diet’ and ‘I always adhere to my diet’ at each end [36,37,38,39,40,41]. Nonetheless, no quality control or standard is available for dietetic review due to local diets and habits targeting a specific structured interview related to the quality of the diet. To date, there is a lack of studies on GFD review outcomes in different countries, and there is no evidence that a proper review can replace other tools (e.g., biopsy) to predict mucosal damage. Moreover, individuals tend to inaccurately report their adherence level, whether intentionally or not, so that dietetic review could be subjective and not identify involuntary infringements [42,43].

### 2.3. Biopsies

Biopsies are a key component for diagnosis, and sometimes it is also necessary for monitoring. During upper intestinal endoscopy at least one biopsy samples should be taken from the bulb and, at least four biopsies, from the second or third portion of the duodenum. Typical features of CD include an increase of intraepithelial lymphocytes (IELs), elongation of the crypts, and partial to total villous atrophy. Therefore, a complete description of the orientation, number of IELs, the presence or not of normal villi or degree of atrophy and crypt elongation, and grading according to the Marsh-Oberhuber classification must appear in the pathology report [44]. The original Marsh classification [45] based on normal mucosa (Marsh 0) to the appearance of lymphocytic infiltration (Marsh 1), crypt hyperplasia (Marsh 2), and different levels of villous atrophy (Marsh 3a–c) results may be subjective. In the last years the modifications made by Oberhuber [46], Corazza and Villanacci [47], Ensari et al. [48], Villanacci [49], or by Ensari [50] have been proposed as more objective and practical. Both classifications made by Corazza and Villanacci [47] and by Ensari [48] are practical and have proven to be useful with good specificity and sensitivity, discriminating latent CD from patients with normal mucosa and identifying those at an early stage. Moreover, Villanacci [51] points out the advantage of including the term of “microscopic enteritis” as a separate histopathology diagnosis. Peña [52] provided a very useful tabbed comparison between different classifications, allowing for compilation and analysis of data for public health.

Classifications based on objective quantitative morphological parameters, such as measurements of height-to-crypt-depth ratio and inflammatory variables, such as the density of IELs with a proper protocol, have been welcome. Taavela et al. [53] evaluated these quantitative morphological and inflammatory variables in the assessment of different degrees of damage to provide cut-off values to be employed in routine clinical practice in CD. The subtyping of the IELs by histological and immunological research and the utilization of flow cytometry and/or immunohistochemistry to the study of IELs have been pointed out to be of paramount importance in the diagnosis and follow-up of CD [54,55,56]. The ratio of the upper normal limit of IELs in the proximal small intestine used as a criterion of the Marsh-Oberhuber classification for gluten sensitivity was established in 40 IELs per 100 epithelial cells (EC) [57]. However, recent studies have observed the upper normal limit in the proximal small intestine to be as low as 20 IEL/100 EC at the tips of five villi on hematoxylin-eosin stained sections and 25 IEL/100 EC with immunohistochemistry by using more thinly cut sections of 3 µm and 4 µm and CD3 immunohistochemistry [58]. 

Despite the use of endoscopies to collect biopsies and assess mucosal healing being the gold standard, it is an invasive, expensive, and impractical procedure for frequent monitoring of disease activity or severity [59]. There are a proportion of cases difficult to monitor and evaluate with biopsy because they have mild histological changes or there is a lack of concordance between serology and histology. Therefore, the idea of re-assessing the emphasis on the biopsy as a gold standard in the follow up of CD, in light of available less invasive tests, is a welcoming one. It has been reported that complete recovery of duodenal mucosa extends over one year, with IELs frequent even 2–5 years after celiac diagnosis [60]. Some experts do not routinely perform a follow-up biopsy in asymptomatic patients with negative serology and good adherence [61]. However, inflammation of the intestinal mucosa can occur long before the development of clinical signs or a rise in antibody titers following a gluten challenge. On the other hand, in NRCD patients with absence of clinical response to a strict GFD should prompt repeat biopsy and additional investigations [62]. Therefore, there is no consensus on the role of follow-up biopsies [18,44].

### 2.4. Serological Tests

Anti-gliadin antibodies (AGA) were the first to be used as screening tool for the disease [63]. Since that time, serologic testing advanced from an adjunctive aid in diagnosis to an integral component of diagnosis, management, and clinical research. Highly sensitive and specific tests, including tTG, endomysial antibodies (EMA), and deamidated gliadin peptide (DGP) antibodies, have been identified in optimizing diagnostics and screening studies [44]. Indeed, for all individuals in whom CD is being considered, serological blood testing should be the initial step in evaluation [62,64]. Despite these advances and the overall laudable test performance of EMA, tTG, and DGP for CD diagnosis, current testing still is subject to a number of important limitations that are important for both clinicians and researchers to recognize. One of the most practical issues currently faced by clinicians is the diversity of available testing platforms, many of which have different cutoff levels, dynamic ranges, and overall test performance. This issue, which has gone largely unaddressed, can be a major impediment to both patient care and research when values are not comparable between providers or between studies. Furthermore, monitoring disease activity in treated CD patients remains a challenge [64]. Although the CD antibody tests show a high accuracy for selecting patients needing a diagnostic biopsy, these tests do not seem to be reliable after diagnosis as the autoantibody titers do not correlate well with histological findings or symptoms in CD patients on a GFD [34,65,66,67,68,69,70]. This may be due to their long half-life and the fact that these titers reflect the immune response rather than direct intestinal damage. IgA- and IgG-class tests can often take 6–24 months to decrease after the antigen source has been eliminated from the diet. In addition, it is important to note that serological tests are not adequate enough to show positive results in patients submitted to small or infrequent exposures to gluten [61]. 

### 2.5. Other Markers

Other studies suggested as suitable diet monitoring markers the permeability test, fecal calprotectine, REG Iα or, recently, plasma total alkylresorcinols [71,72,73,74]. However, several studies have reported these tests not being only specific to CD but also of limited efficacy in the diagnosis of uncomplicated CD [75,76,77]. Two other markers are intestinal-fatty acid binding protein (I-FABP), a marker reflecting enterocyte damage, and citrulline, a marker for functional enterocyte mass [64], but they are not specific for CD, so they do not discriminate between a celiac relapse or other gastrointestinal disorders in the patient.

Autoantibodies against pancreatic secretory-granule membrane glycoprotein 2 (GP2), especially of IgA isotype, have been demonstrated in patients with Crohn’s disease and, recently, also with CD. In CD patients with anti-GP2 antibody positivity, this marker could be used as indicator for intestinal inflammation and for follow-up. However, CD should be differentiated from Crohn’s disease by parallel testing of CD-specific EMA or anti-tTG [78,79].

Recently, Ryan et al. [80] reviewed the metabolomics associated to the diagnosis and prognosis in CD as a significant potential tool. The identification of three main components (malabsorption, energy metabolism, and alterations of gut microbiota) in matrices, such as sera, urine, and feces, has been of particular interest in the metabolome of CD. Different compounds related to malabsorption (decreased levels of amino acids, lipids, pyruvate, and choline in the sera of celiac patients), other components were related to energy metabolism (higher levels of glucose and 3-hydroxybutyric acid in sera) and, thirdly, those related to alterations of gut microbiota and/or intestinal permeability as higher levels of indoxyl sulfate, meta-[hydroxyphenyl] propionic acid, and phenylacetylglycine in urines [81,82]. 

### 2.6. Detection of Gluten Immunogenic Peptides (GIP)

The above tests to monitoring GFD compliance only evaluate the consequences of GFD transgressions. Moreover, they are unable to detect occasional gluten exposure that may impede total gut mucosa recovery in the celiac patient [34,65,66,67,83,84,85,86,87,88]. In this respect, it is noted that a diet with zero gluten intake is impossible due to its ubiquity; thus, a minimal level of gluten contamination is present in the daily diet. In fact, total daily gluten consumption that could be critical for most CD patients is of <50 mg gluten [89], and some patients need as little as 10 mg of daily gluten to promote development of intestinal mucosal abnormalities [90]. Therefore, there is a need for accurate, non-invasive tools for managing patients to show gluten intake and avoid the harmful aftermaths.

CD is triggered by the certain gluten immunogenic peptides (GIP) are resistant to gastrointestinal digestion and can interact with the immune system of patients with CD to trigger an autoimmune response against tTG and other antigens. Shan et al. [91] showed by in vitro and in vivo studies in rats and humans that a 33-mer peptide from α2-gliadin is stable toward breakdown by all gastric, pancreatic, and intestinal brush border membrane endoproteases. This peptide was identified as the primary initiator of the inflammatory response to gluten in patients with CD [91]. Toward the assessment of toxicity and GIP in foods for celiac patients, G12 and A1 monoclonal antibodies (moAbs) were obtained against 33-mer peptides. The reactivity of these antibodies was correlated with the potential immunotoxicity of the proteins analyzed and they proved to be useful in studies about the enzymatic detoxification of gluten [92,93]. These antibodies displayed a great sensitivity to toxic peptides (besides the 33-mer peptide) from wheat, rye, barley, and varieties of oats [92,93]. A sandwich enzyme-linked immunosorbent assay (ELISA) based on G12 and A1 moAbs gave very promising results for gluten analysis across a range of samples. This method had a limit of detection of 0.6 ppm gluten, 1/3 of the concentration obtained by other methods described to date. Similarly, a rapid test for the detection of gluten in solid food, drinks, and on surfaces using G12 moAb lateral flow devices (LFD) or dipsticks [94,95], as well as a competitive ELISA method were also developed for the detection of toxic gluten peptides in hydrolyzed foods [94,96]. More interesting, G12 immunodepletion experiments with hydrolyzed gliadin from beers showed that this moAb recognize those with the highest immunoactivity for celiac patients, this is a significant advance in the detection of the inmunoactive gluten content in the gluteome [97]. Based on these methodologies, new tools have been proposed for monitoring the GFD by determining GIP in human samples.

#### 2.6.1. Feces

Immunoassays with G12 moAb showed that >30% of the inmunoreactive gliadin peptides persisted intact after hydrolysis during in vitro simulated gastrointestinal digestion [98]. Based on these findings, Comino et al. [98] described a novel method to monitor the GFD by detection of GIP in feces by using the G12 antibody [99]. This study supports the resistance of the 33-mer to in vitro peptic-tryptic-chymotryptic hydrolysis; and, most significantly, it was shown that toxic epitopes of gluten are measurable by moAbs in the feces of normal subjects and CD patients receiving a gluten-containing diet. The resistance of gluten peptides to gastrointestinal digestion, in particular peptides related to the immunotoxic 33-mer peptide, ensures that an important part of the ingested gluten is eliminated in feces. Consequently, the recovery of measurable levels of the immunotoxic fraction in feces suggests that gluten has passed through the digestive tract and, therefore, that gluten has been ingested. GIP were detected in the feces of healthy individuals and CD patients receiving gluten-containing diets, and GIP disappeared when a GFD was introduced [100]. With diets that contained variable quantities of gluten, GIP excretion was proportional to the amount ingested. These tests could also detect differences when, being in GFD, subjects were challenged with known amounts of oral gluten [98].

**Figure 1:**
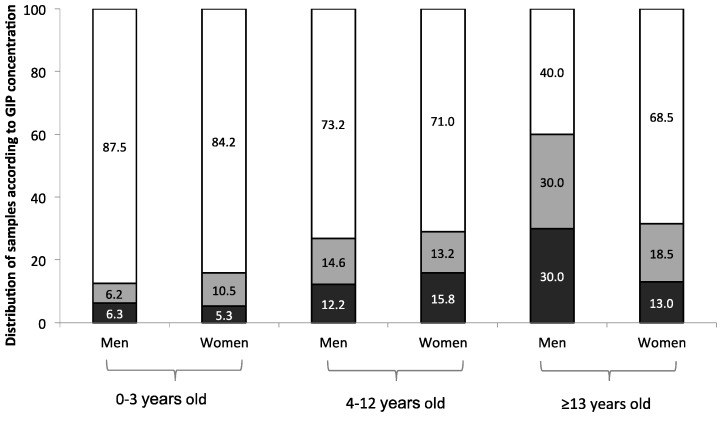
Percentage distribution of celiac patients by GIP content in feces by age and sex. GIP, gluten immunogenic peptides. GIP positive (>0.30 µg GIP/g feces, black bar), weak positive (0.16–0.30 µg GIP/g feces, grey bar), and negative (<0.16 µg GIP/g feces, white bar).

A recent study has shown the clinical usefulness of this new method of measuring fecal GIP as a marker of adherence to GFD [43]. A multicenter clinical trial prospectively examined the compliance to the GFD of both celiac children and adults. Furthermore, the response rate to GFD was evaluated by dietary questionnaire, celiac serology, and clinical response. Correlations between fecal GIP and traditional methods to monitoring the GFD were investigated. The majority (85.7%) of celiac children between zero and three years of age had feces negative for GIP, with only 14.3% showing levels above the limit of quantification. The proportion of celiac patients with feces positive for GIP increased to 27.8% in children between four and 12 years of age. Among those ≥13 years old, the proportion rose up to 39.2% with positive GIP. When further stratified by gender, adherence to the GFD was found to be closely related to the patient’s gender in certain age groups. More males ≥13-years old had positive GIP feces compared with females in the same age group (60% vs. 31.5%, *p* = 0.034), indicating higher numbers of dietary transgressions among males than in their female peers (Figure 1). Although no overall significant differences between the percentage of GIP-positive feces in celiac patients and the duration of the GFD were observed, the patients who had been on the GFD for a longer period of time showed higher rates of noncompliance. No significant association was found between GIP levels in celiac patients and history of CD in their first- or second-degree relatives.

Comino et al. [43] also showed no association between fecal GIP and dietary questionnaires or anti-tTG antibodies. However, association was detected between GIP and anti-DGP antibodies, although 46 of the 53 GIP feces-positive patients were negative for anti-DGP. Detection of gluten peptides in feces reveals limitations of traditional methods for monitoring GFD in celiac patients. Fecal GIP analysis is an accurate and noninvasive method that enables a direct and quantitative assessment of gluten exposure early after ingestion. Therefore, these methods could aid in the diagnosis and clinical management of NRCD and RCD [43].

#### 2.6.2. Urine

**Figure 2:**
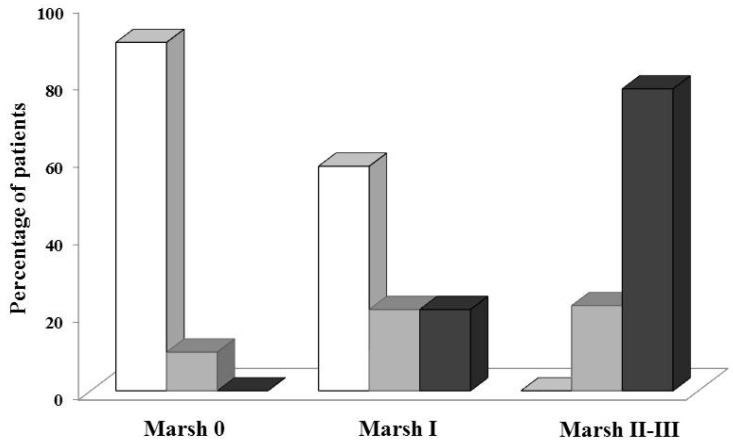
Presence of GIP in urine of adult CD patients and correlation with their small bowel histology. Severity of mucosal lesion (Marsh I–III) and histological appearance determined by the Marsh scale. GIP negative (white bar), absence of GIP in urine; GIP weak positive (grey bar), visual presence of GIP not quantifiable in urine (>LDT < QL); GIP positive (black bar), presence of GIP visible and quantifiable in urine (>QL). *p* = 0.0007 (Fisher’s exact test). Values are expressed as the percentage of patients. CD, celiac disease; GIP, gluten immunogenic peptides; LDT, limit of technique detection; QL, quantification limit. Modified according to Moreno et al. [103].

A proportional fraction of the GIP absorbed in the gastrointestinal tract makes it to the circulation and is excreted in urine [101,102]. The methodology proposed by Moreno et al. [103] based on urine gluten testing may be useful in clinical practice as a monitoring tool to follow-up the compliance of GFD. Clinical assays in urine based on LFD are used in many diseases. Coupling a reader to the LFD in urine of CD patients could provide a quantitative measurement of dietary infringement, providing significant advantages in the management of GFD. A positive correlation between the amount of ingested gluten and GIP detected in human urine samples has been demonstrated [103]. It has been determined in urine the low intake of gluten in processed bread, >25 mg corresponding to the lower limit to exert damage to most celiac patients. GIP were detected in urine samples 6–48 h after gluten intake. The methodology demonstrated the high level of noncompliance in patients with CD who had supposedly consumed long-term GFD through the presence of GIP (48% and 45% in adults and children, respectively). These results were consistent with reports showing that ~30%–50% continue with mucosal atrophy in CD patients despite following a GFD [5,104,105,106]. More interestingly, a direct correlation was demonstrated between the absence of GIP in urine and healing of the gut intestinal epithelium (Figure 2). Furthermore, 100% of the adult patients with higher damage in the epithelia (Marsh II/III), according to the histological analysis, had GIP in urine. In accordance with other above-mentioned studies [34,67,85], this study confirmed the poor correlation of serological tests with mucosal healing, as well as the shortcomings of the dietary history questionnaires to assess GFD compliance.

The development of point-of care devices for an accurate, simple, and efficient GFD monitoring motived the creation of the highly-sensitive surface plasmon resonance biosensor for the detection of GIP in urine [107]. The easy-to-handle samples, such as urine and user-friendly biosensors could be suitable for the portable and simple devices for the GFD compliance of celiac patients. Soler et al. [107] demonstrated that the sensing methodology enables rapid and label-free quantification of the GIP in urine by using G12 moAb, reaching a limit of detection of 0.33 ng/mL. This study also clearly differed gluten consumers from non-consumers by measuring several urine samples from both healthy (normal diet) and celiac subjects (GFD). Therefore, biosensors offer significant advantages over conventional techniques enabling biochemical analysis with excellent reproducibility and high sensitivity in a matter of minutes.

## 3. Conclusions and Future Directions

It is often difficult to evaluate compliance with GFD. Persistent gluten exposure is usually unintentional. Exposure may occur no matter how careful a patient is, due to cross-contamination or simple lack of knowledge regarding the diet. Serum markers for CD play an important role in CD management (mostly tTG); however, the evidence suggests that it is not sensitive enough to detect occasional significant dietary transgressions that impede mucosa healing. There is no agreement on the role of follow-up biopsies and it is an invasive procedure, expensive and impractical for frequent monitoring of this disease. Moreover, an issue to address is the lack of studies comparing diagnostic efficacy of biomarkers with histology in patient follow-up. Notwithstanding, the need for non-invasive approaches to monitor CD is certainly warranted. Some studies related to metabolomics and other recent markers can measure the consequences of dietary transgressions, but they cannot show direct gluten intake, and they are not specific for CD. The incorporation of simple immunological assays based on GIP analysis in human samples could resolve some scientific and clinical problems in CD management such as (i) detection of inadvertent lapses after appearance of acute symptoms; (ii) in celiac patients, with or without symptoms, and patients with non-celiac gluten sensitivity; (iii) non-compliance of the GFD before any anatomic damage; (iv) to prove gluten intake during CD diagnosis; (v) examining the adherence to the GFD in the initial period after diagnosis when patients are less familiar with this diet; and (vi) the accurate diagnosis and management of the diet in NRCD and RCD. Therefore, these tests may prevent a potentially invasive and extensive medical analysis to assess the cause of the ongoing symptoms of celiac patients.
